# Association Between Sense of Coherence and Health Outcomes at 10 and 20 Years Follow-Up: A Population-Based Longitudinal Study in Germany

**DOI:** 10.3389/fpubh.2021.739394

**Published:** 2021-12-10

**Authors:** Anna Dziuba, Janina Krell-Roesch, Steffen C. E. Schmidt, Klaus Bös, Alexander Woll

**Affiliations:** Institute of Sports and Sports Science, Karlsruhe Institute of Technology, Karlsruhe, Germany

**Keywords:** salutogenesis, salutogenic, sense of coherence, antonovsky, health

## Abstract

**Background:** The sense of coherence (SOC) is reported to influence health, but health may also have an impact on SOC. The objective of this study was to examine the longitudinal associations between SOC and selected self-reported and physician-assessed health outcomes over a period of 10 and 20 years and to determine the predominant direction of the associations.

**Methods:** We conducted a population-based, longitudinal study, involving 392 participants (188 females and 204 males; mean age 43.01 years) who were followed for a median of 10 and 18 years. Analyses of variance were carried out to examine the longitudinal associations between SOC at baseline and health outcomes (i.e., self-rated health status, SHS; physical health status assessed by a physician, PHS; self-reported satisfaction with life, SWL) at follow-ups. The direction of associations was examined using a cross-lagged model on correlation coefficients.

**Results:** There were significant group effects for SOC at baseline on SHS at 20-year follow-up (*F* = 4.09, *p* = 0.018, η*p*^2^ = 0.041), as well as on SWL at 10-year (*F* = 12.67, *p* < 0.01, η*p*^2^ = 0.072) and at 20-year follow-up (*F* = 8.09, *p* < 0.1, η*p*^2^ = 0.069). SHS (*r* = 0.238, *p* < 0.01), PHS (*r* = −0.140, *p* < 0.05) and SWL (*r* = 0.400, *p* < 0.01) predicted SOC at 10-year follow-up stronger than vice versa. The direction of associations between SOC and health parameters at 20-year follow-up was less consistent.

**Conclusions:** The long-term associations between SOC and self-reported and physician-assessed health may be reciprocal in community-dwelling adults. More research is needed to examine the predictive power of health on SOC and whether interventions targeted at improving health parameters, may impact SOC.

## Introduction

Already 50 years have passed since Antonovsky introduced a salutogenic model focusing on the origins of health and well-being (1). This model seeks to explain why some individuals are capable of maintaining and even improving their health in stressful life situations. The main concept behind the salutogenic approach is the sense of coherence (SOC). Antonovsky described the SOC as a global orientation to view one's own life as structured, manageable, and meaningful (2). SOC can also be understood as the capacity of a person to cope with stressors in daily life by identifying and using their generalized resistance resources (GRR, e.g., intelligence, ego identity, social support, cultural and preventive health orientation) to maintain and strengthen their health (3, 4). SOC is influenced by life experience whereas the GRRs provide an individual with sets of meaningful life prerequisites (5). Antonovsky emphasized that the SOC concept is a dispositional orientation rather than a personality trait or a coping strategy (6). Furthermore, the SOC represents a salutogenic factor, actively promoting and facilitating health (7).

Numerous empirical studies have shown that the SOC is associated with positive subjective well-being, mental health, and quality of life [for recent systematic reviews, see (8)] (9, 10). Furthermore, the SOC is associated with lower severity of symptoms of anxiety and depression (3, 5, 11). The correlation with health in general ranges from moderate to high, explaining up to 66% of the variance in SOC depending on the assessment tools used (12). The remaining variance is accounted for by other factors such as age, social support, and education. In addition, the SOC is also associated with physical health, but reported correlations are usually significantly lower and less consistent. This may indicate that the SOC mainly comprises an individual's mental, social, and spiritual resources to cope with the challenges of life. Schumacher et al. also assumed that the SOC does not directly affect physical health, but only indirectly and mediated through coping behavior in stressful situations (13). To date, only a few longitudinal studies with an observation time, which is sufficient to analyze the predictive associations of SOC on health and vice versa throughout a lifespan exist (14–19). The SOC appears to have relatively high predictability, both in a short term (i.e., several months) and a long term (i.e., several years) perspective (8). Länsimies et al. (9) reported that a higher SOC in adolescents is a predictor of various health outcomes (e.g., quality of life, health behavior, and mental health) in adulthood. However, conflicting results have been reported where SOC was not associated with health outcomes in adults. (3). It has also been postulated that the relationship between SOC and health could rather be reciprocal, i.e., SOC potentially influences health, but health may also have an impact on SOC (2, 8).

The aim of the present study was to examine the longitudinal associations between SOC and three self-reported and physician-assessed health outcomes in community-dwelling adults aged ≥33 years. This included (a) examining the associations between SOC at baseline (as predictor variable) and health outcomes at follow-ups (as outcome variables), and (b) identifying the predominant directions of associations between SOC and health outcomes through cross-lagged analyses. We hypothesized that SOC at baseline would be associated with more favorable health outcomes at follow-up, but that nowadays the current or previous health status may be a better predictor for SOC than vice versa. This implies that the predominant direction of causality between SOC and health parameters has shifted in today's relatively healthy society. This knowledge might be crucial for current interventions to prevent the potential multiplication of negative life events (e.g., accidents, the manifestation of chronic disease) as they influence both current health status and SOC as a potential future protective factor.

## Materials and Methods

### Study Population

The study was conducted in the setting of a community-based, longitudinal research in southern Germany (20). Data from five measurement waves carried out in 1992, 1997, 2002, 2010, and 2015 were used. Participants aged ≥33 years were randomly selected from the residents' registration offices in Bad Schönborn, Germany. All participants gave written informed consent before taking part in the research. Participation was voluntary. The study was approved by a scientific advisory council, the Schettler Clinic, Bad Schönborn, Germany as well as the ethics committee of the Karlsruhe Institute of Technology (KIT). We strictly followed ethical guidelines from the German Psychological Society. All data was analyzed anonymously.

Overall, 392 individuals (188 females and 204 males) aged 33–67 years participated in this study. The response rate of the initial sample in 1992 was 56%. For the initial sample, individuals from five age strata (i.e., 35, 40, 45, 50, and 55 ± 2 years) were invited for participation. A non-responder telephone interview showed no significant differences in socioeconomic status (SES), physical health status, and physical activity between participants and invited non-participants except for migration background (21). In each subsequent wave, additional participants from 28 to 38 years of age were recruited to compensate for dropouts (22). The sample characteristics concerning SES are representative of a rural community in Germany (23).

The sample for the 10-year follow-up analysis on the associations between SOC and health outcomes consists of 349 individuals (166 females and 183 males) aged 33-67 years; and for the 20-year comparison, the sample consists of 240 individuals (113 females and 127 males) aged 33–62 years. The first-ever participation of an individual in the study was considered the baseline measurement (i.e., 1992, 1997, 2002). If participants had valid data from study participation 10 or 20 years after the baseline measurement then these data were considered for the 10-respectively, 20-year analyses. Missing data led to exclusion from the study.

### Measures

Details on study procedures and measures have been published elsewhere (21). In every study year, the data assessment took place between May and June.

**Socioeconomic Status (SES)**. Based on methods for social structure analyses (24), participants were grouped into four SES categories using self-reported information about formal education and professional status. If participants were not working or data was missing, the professional status of the spouse or partner was used. The four SES categories are low, low/medium, medium/high, and high.

**Physical activity**. Participants were classified into being physically active or inactive based on two questions, i.e., “Do you engage in sports?” with response options yes or no, and “What type of sports do you do?” Individuals with missing information were excluded from the distribution.

**Sense of coherence (SOC)**. The authorized German translation of the 13-item version of the Orientation to Life Questionnaire (SOC-13) was used (2). The answers are provided on a seven-point response scale ranging from 1 point to 7 points, with 1 and 7 indicating extreme feelings about questions (and statements) about how one's life is experienced (e.g., “when you talk to people, do you have the feeling that they do not understand you?” is scored from 1 = “never have this feeling” to 7 = “always have this feeling”). A total score for all 13 items of the SOC-13 was computed, ranging between 13 and 91 points. Higher values indicate a stronger SOC. Participants who had not answered all 13 questions were excluded. The SOC-13 has proven to be a reliable, valid, and cross-culturally applicable instrument that measures how individuals manage stressful situations and maintain their well-being (12, 13, 25, 26).

**Self-rated health status (SHS)**. All participants were asked to rate their health status by answering the following 5 questions: “how do you describe your health status?,” “how does your current health status affect your job performance?,” “how does your current health status affect your leisure activities?,” “how do you describe your health status compared to others of your age and gender?” and “has your health status changed in the last 5 years?” Responses were given on a scale of 1–5 points, where 1 indicates a very low health status and 5 reflects a very high health status (e.g., “how do you describe your health status” is scored from 1 = “very poor” to 5 = “very good”). Missing values were imputed by the mean value of the participant. An overall score was calculated for the self-rated health status, ranging between 5 and 25 points with a higher score indicating a better self-rated health status.

**Physical health status (PHS)**. Physical health status was assessed based on a thorough medical examination performed by a licensed physician. If any part of the examination could not be performed, the physician provided a subjective assessment to prevent missing values. After the comprehensive examination, the physician made a diagnosis of 0 = “no limitations,” 1 = “minor limitations, not impacting daily life,” 2 = “limitations, impacting daily life,” and 3 = “major limitations, heavily impacting daily life” concerning orthopedic, neurological, and cardiovascular health. An overall score was created of the three diagnoses, ranging from 0 to 9 points. A higher score suggests more limitations.

**Satisfaction with life (SWL)**. Satisfaction with life in general was measured by a single question “how satisfied are you with your life?,” with response options ranging from 1 = “very dissatisfied” to 5 = “very satisfied.”

### Data Analysis

Demographics of the study samples at baseline were calculated separately for the 10- and 20-year analyses. For categorical variables (e.g., sex, physically active, SES), we calculated number (*N*) and percentage (%), for continuous variables (e.g., age), we calculated means (M) and standard deviations (SD). To test for changes between baseline and 10- and 20-year follow-up, respectively, we ran paired *t*-tests. Analyses of variance (ANOVA) controlled for age and sex were carried out to examine the longitudinal associations between SOC at baseline and the three different health outcomes (i.e., SHS, PHS, and SWL) at 10 and 20 years of follow-up, respectively. To assess the differences between individuals with different SOC statuses, individuals are frequently divided into different percentiles based on their SOC scores (27–30). In line with these studies, the SOC variable at baseline was divided into three subgroups at the 20% and 80%-percentiles. Participants with a score of 13-54 points were considered as having a low SOC (i.e., 20% of participants with lowest SOC), 55–64 points indicated a moderate SOC, and 65-91 points indicated a high SOC (i.e., 20% of participants with highest SOC). These SOC groups were entered into the models as between-subjects factors whereas the repeated measures were considered as within-subjects factors.

In addition, a cross-lagged model on correlation coefficients to ascertain the direction of the associations between SOC and health outcomes was performed. Although cross-lagged designs should not be interpreted as definitive proof of causality, they can be an indication of the predominant direction of effects over time (24, 31, 32). In a cross-lagged design two variables X and Y are measured at two time points, resulting in three types of relations: within-time or synchronous relations indicating the cross-sectional association between X1 and Y1, or X2 and Y2; autoregressive or stability relations indicating the longitudinal prediction of X2 by X1, orY2 by Y1; and cross-lagged relations indicating the longitudinal prediction of Y2 by X1, or X2 by Y1 (7). Pearson's product moment correlation coefficients were calculated for the analysis of the partial correlations. The effect sizes applied in this study follow Cohen's recommendation for behavioral sciences: *r* = 0.1 small; *r* = 0.3, medium; and *r* = 0.5, large (33). Sex and age at baseline were included in these models as covariates.

A *p*-value < 0.05 was considered to be statistically significant. The data were analyzed using SPSS for Windows, version 26 (Chicago, Illinois, USA).

## Results

### Demographic Characteristics

Descriptive statistics of the samples at baseline for the 10- and 20-year follow-up analyses, as well as of SOC, SHS, PHS, and SWL variables at baseline and follow-up are shown in Table 1. The distributions of sex, age, SES, and the number of physically active persons are very similar in both study samples.

**Table 1:** Demographics of the samples for 10- and 20-year follow-up analyses.

**Variables**	**All participants *n* = 392**	**10-year follow-up** ***n*** **=** **349**		**20-year follow-up** ***n*** **=** **240**	
Sex, *n* (%)			
Female	188 (48)	166 (48)		113 (47)	
Male	204 (52)	183 (52)		127 (53)	
Age* in years, M (SD)	43.01 (8.19)	43.00 (8.24)		42.54 (7.71)	
SES*, *n* (%)			
Low	25 (6.5)	24 (7.0)		11 (4.7)	
Low/medium	97 (25.2)	83 (24.1)		61 (25.8)	
Medium/high	147 (37.5)	134 (39.0)		83 (35.2)	
High	116 (30.1)	103 (29.9)		81 (34.3)	
Physically active*, *n* (%)	251 (64.5)	223 (64.5)		166 (69.5)	
					
		**Baseline**	**Follow-up**	**Baseline**	**Follow-up**
					
SOC total score, M (SD)		59.90 (6.94)	68.56 (10.28)	59.19 (6.03)	68.28 (10.58)
SOC groups, *n* (%)					
Low		68 (19.7)	31 (8.9)	44 (18.4)	21 (8.8)
Moderate		203 (58.7)	67 (19.3)	154 (64.4)	44 (18.3)
High		75 (21.7)	250 (71.8)	41 (17.2)	175 (72.9)
SHS total score, M (SD)		17.35 (2.60)	16.94 (3.25)	17.36 (2.58)	16.86 (3.14)
PHS total score, M (SD)		1.60 (1.38)	1.35 (1.80)	1.63 (1.32)	2.17 (1.80)
SWL total score, M (SD)		4.11 (0.59)	4.12 (0.63)	4.13 (0.55)	4.17 (0.63)

SES, socioeconomic status; SOC, sense of coherence: 13–91 points; SHS, self-rated health status: 5–25 points; PHS, physical health status: 0–9 points; SWL, satisfaction with life: 1–5 points ^*^at baseline.

In the sample for the 10-year analyses, SOC total score showed a significant increase from baseline to follow-up (*p* < 0.01). Similarly, when comparing the SOC groups, the number of participants in low and medium SOC groups decreased from baseline to follow-up, whereas the number of participants in the high SOC group increased from baseline to follow-up. With regard to the different health-related outcomes, both SHS total score (i.e., subjective) and PHS total score (i.e., objective) showed a minimal decrease (SHS: *p* = 0.014; PHS: *p* = 0.228) from baseline to follow-up. However, in general, participants reported low limitations at both baseline and follow-up. Finally, participants reported a high SWL total score both at baseline and at follow-up. There was only a slight increase in mean scores from baseline to follow-up (*p* = 0.478).

In the sample for the 20-year analyses, SOC mean total score increased significantly from baseline to follow-up (*p* < 0.01). However, the health status of participants declined, i.e., SHS showed a small decrease (*p* = 0.106) between baseline and follow-up, whereas PHS increased significantly between baseline and follow-up (*p* < 0.01); and SWL mean total score increased slightly (*p* = 0.207).

### Longitudinal Associations Between SOC and Health Outcomes

The results of the ANOVA are shown in Table 2, separately for the 10-year and 20-year follow-up samples.

**Table 2:** ANOVA on longitudinal associations between SOC groups at baseline and SHS, PHS and SWL at 10- and 20-year follow-up.

		**10 years**			**20 years**		
		***n***	**Baseline**	**Follow-up**	***n***	**Baseline**	**Follow-up**
**SHS M (SD) score**							
SOC	Low	56	16.77 (2.79)	16.54 (3.38)	35	16.34 (2.78)	16.26 (2.99)
	Moderate	175	17.66 (2.37)	16.83 (3.38)	123	17.47 (2.57)	16.92 (3.41)
	High	66	17.53 (2.84)	17.44 (2.75)	37	17.70 (2.50)	17.08 (2.17)
	∑	297	17.46 (2.58)	16.91 (3.22)	195	17.31 (2.62)	16.83 (3.14)
Time		*F*_(1, 292)_ = 0.61, *p* = 0.436, *ηp^2^* = 0.002			*F*_(1, 190)_ = 0.31, *p* = 0.577, *ηp^2^* = 0.002		
Group		*F*_(2, 292)_ = 2.79, *p* = 0.062, *ηp^2^* = 0.019			*F*_(2, 190)_ = 4.09, *p* = 0.018, *ηp^2^* = 0.041		
Time*group		*F*_*F*(2, 292)_ = 1.50, *p* = 0.226, *ηp^2^* = 0.010			*F*_(2, 190)_ = 0.13, *p* = 0.878, *ηp^2^* = 0.001		
**PHS M (SD) score**							
SOC	Low	54	1.33 (1.30)	0.96 (1.50)	42	1.29 (1.09)	1.79 (1.76)
	Moderate	170	1.49 (1.35)	1.51 (1.95)	144	1.68 (1.39)	2.23 (1.83)
	High	62	1.53 (1.45)	1.34 (1.65)	38	1.61 (1.22)	2.42 (1.72)
	∑	286	1.47 (1.36)	1.37 (1.82)	224	1.59 (1.31)	2.18 (1.81)
Time		*F*_(1, 281)_ = 0.04, *p* = 0.852, *ηp^2^* = 0.000			*F*_(1, 219)_ = 1.42, *p* = 0.234, *ηp^2^* = 0.006		
Group		*F*_(2, 281)_ = 1.44, *p* = 0.239, *ηp^2^* = 0.010			*F*_(2, 219)_ = 0.41, *p* = 0.663, *ηp^2^* = 0.004		
Time*group		*F*_(2, 281)_ = 1.02, *p* = 0.361, *ηp^2^* = 0.007			*F*_(2, 219)_ = 0.25, *p* = 0.779, *ηp^2^* = 0.002		
**SWL M (SD) score**							
SOC	Low	63	3.78 (0.77)	3.92 (0.68)	42	3.90 (0.69)	4.02 (0.64)
	Moderate	194	4.16 (0.48)	4.19 (0.56)	143	4.14 (0.48)	4.17 (0.63)
	High	73	4.25 (0.55)	4.15 (0.74)	40	4.30 (0.52)	4.38 (0.54)
	∑	330	4.11 (0.58)	4.13 (0.64)	225	4.12 (0.55)	4.18 (0.62)
Time		*F*_(1, 325)_ = 1.33, *p* = 0.715, *ηp^2^* = 0.000			*F*_(1, 220)_ = 0.28, *p* = 0.598, *ηp^2^* = 0.001		
Group		*F*_(2, 325)_ = 12.67, *p* < 0.01, *ηp^2^* = 0.072			*F*_(2, 220)_ = 8.09, *p* < 0.01, *ηp^2^* = 0.069		
Time*group		*F*_(2, 325)_ = 2.24, *p* = 0.108, *ηp^2^* = 0.014			*F*_(2, 220)_ = 0.43, *p* = 0.654, *ηp^2^* = 0.004		

*SOC, sense of coherence; SHS, self-rated health status; PHS, physical health status; SWL, satisfaction with life; controlled for sex & age; p indicates statistical significance; ηp^2^ = partial eta squared to indicate effect size; total n changes as information on various health outcomes was not available on all participants (missing data)*.

Self-rated health status (SHS): Participants reported lower SHS scores at follow-up compared to baseline across all SOC groups in the 10-year model. At baseline, the moderate SOC group had the highest SHS. SHS at 10-year follow-up was highest in participants with high SOC at baseline. However, no significant time, group, or interaction effect could be found. The trend was similar in the 20-year model, i.e., participants with higher SOC at baseline reported higher SHS at both baseline and follow-up. Of note, the mean SHS total score decreased between baseline and 20-year follow-up across all SOC groups. A significant group effect was found (*F* = 4.09, *p* = 0.18, η*p*^2^ = 0.041).

Physical health status (PHS): Participants with moderate and high SOC at baseline and follow-up showed numerically higher mean PHS scores, albeit not significant. The results in the 20-year model differ from those in the 10-year model such that across all baseline SOC groups, PHS increased significantly from baseline to 20-year follow-up. No time, group, or interaction effect was found.

Satisfaction with life (SWL): At 10-year follow-up, participants with a moderate SOC at baseline had the highest SWL total score. SWL mean total score increased between baseline and 10-year follow-up in the low and moderate SOC group and decreased in the high baseline SOC group. For the 10-year model, a significant group effect was observed (*F* = 12.67, *p* < 0.01, η*p*^2^ = 0.072). In the 20-year model, SWL mean total score increased between baseline and follow-up across all baseline SOC groups, and participants with a higher SOC reported a higher SWL at both baseline and 20-year follow-up. A significant group effect was found (*F* = 8.09, *p* = 0.002, η*p*^2^ = 0.069).

### Direction of Associations Between SOC and Health Outcomes

**Figure 1:**
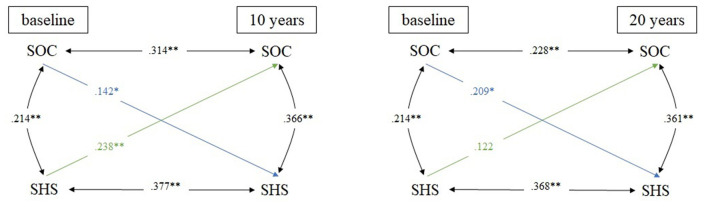
Cross-lagged model linking SOC and SHS in 10- and 20-year comparison; *N* = 395 for all analyses; adjusted for sex & age; *p ≤ 0.05; **p ≤ 0.01; SOC, sense of coherence; SHS, self-rated health status.

**Figure 2:**
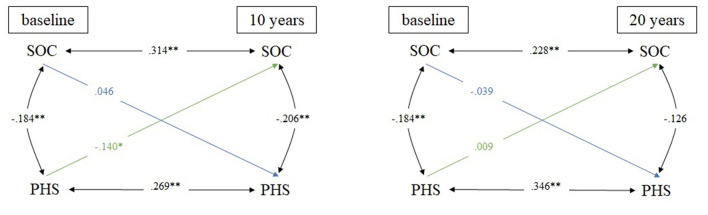
Cross-lagged model linking SOC and PHS in 10- and 20-year comparison; *N* = 395 for all analyses; adjusted for sex & age; *p ≤ 0.05; **p ≤ 0.01; SOC, sense of coherence; PHS, physical health status.

**Figure 3:**
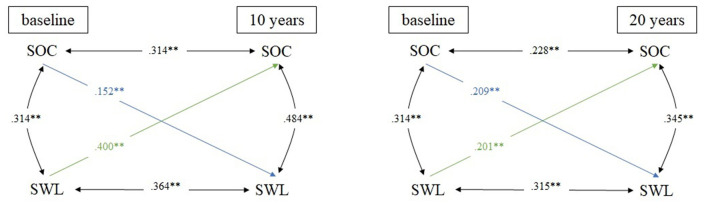
Cross-lagged model linking SOC and SWL in 10- and 20-year comparison; *N* = 395 for all analyses; adjusted for sex & age; **p ≤ 0.01; SOC, sense of coherence; SWL, satisfaction with life.

All cross-lagged partial correlations with sex and age at baseline as covariates are presented in Figures 1–3.

The first cross-lagged model is linking SOC and SHS (Figure 1). In the 10-year comparison, stability coefficients were moderate, ranging from 0.314 (SOC) to 0.377 (SHS). Regarding the cross-lagged relations, both correlation coefficients were small. However, SHS at baseline positively predicted SOC at follow-up more strongly (*r* = 0.238) than vice versa (*r* = 0.142). In the 20-year comparison, the stability relation was small regarding SOC (*r* = 0.228) and moderate regarding SHS (*r* = 0.368). Only a significant small cross-lagged correlation between SOC at baseline and SHS at follow-up (*r* = 0.209) was found. Of note, within-time relations between SOC and SHS were small at baseline (*r* = 0.214) and moderate both at 10-year (*r* = 0.366) and 20-year follow-up (*r* = 0.361).

The second cross-lagged model describes the association between SOC and PHS (Figure 2). Stability coefficients in the 10-year comparison ranged from 0.269 (PHS) to 0.314 (SOC). No significant cross-lagged relationship between SOC at baseline and PHS at follow-up was found, in turn, the correlation coefficient between PHS at baseline and SOC at follow-up was small. Less limitation (i.e., lower PHS scores) at baseline positively predicted SOC at 10-year follow-up (*r* = 0.140). In the 20-year comparison, the stability relation was small regarding SOC (*r* = 0.228) and moderate regarding PHS (*r* = 0.346). No significant cross-lagged relation was found, whereas small within-time relations at all measurement times were observed.

Figure 3 is showing the third cross-lagged model linking SOC and SWL. In the 10-year comparison, moderate stability coefficients were found for both SOC (*r* = 0.314) and SWL (*r* = 0.346). Regarding the cross-lagged correlation, the correlation coefficient was small between SOC at baseline and SWL at follow-up (*r* = 0.152), whereas it was moderate between SHS at baseline and SOC at follow-up (*r* = 0.400). In the 20-year comparison, stability coefficients ranged from 0.228 (SOC) to 0.315 (SWL). The cross-lagged correlations were small for both directions of effects, i.e., a high SOC at baseline positively predicted SWL at 20-year follow-up (*r* = 0.209), and a high SHS at baseline vice versa positively predicted SOC at 20-year follow-up (*r* = 0.201). Within-time coefficients were moderate at all measurement times, ranging from 0.134 (baseline) to 0.484 (10-year follow-up).

## Discussion

With regard to the first research question, we observed that SOC total score increased significantly over 10 and 20 years of follow-up. In the 10-year model, there was no significant change in health status (i.e., SHS, PHS, and SWL), whereas in the 20-year model physical health status decreased significantly regardless of SOC at baseline. Antonovsky (1, 2) assumed SOC to be a health resource that was fundamentally acquired in early childhood and adolescence without any major changes after the age of 30. According to our results and other studies, we assume that SOC develops positively along the entire lifetime (34). Furthermore, our results showed that SOC at baseline appears to have a significant influence on SHS at 20-year-follow-up as well as on SWL at 10- and 20-year follow-up, but not on PHS. Thus, SOC may have a greater impact on subjective health than objective health. These findings are in line with previous longitudinal studies reporting that SOC is associated with positive subjective well-being, mental health, and a lower number of subjective complaints and symptoms of illness (3, 5, 14, 15, 17–19, 35). Suominen and Lindström (36) reported that people who have developed a strong SOC manage disease (i.e., mental, physical, or social) better than those with a weak SOC. This relation was not only found among adults but also among adolescents (7, 37). Furthermore, SOC is strongly related to self-rated health score and possession of health-promoting resources that support the development of a positive subjective state of health (8). Our study adds to the existing body of research by providing preliminary evidence of longitudinal associations between SOC at baseline and better SWL at both 10 and 20 years of follow-up. This relationship supports Antonovsky's view of the SOC as a global life orientation. He assumed that a strong SOC reduces the perceived strain of life (36).

Regarding the cross-sectional associations between SOC and health outcomes, we found positive correlations between SOC at baseline and SHS at both follow-ups, whereas SHS at baseline only predicted SOC at 10-year follow-up. No correlations were found between SOC at baseline and PHS at follow-ups, while PHS at baseline positively predicted SOC at 10-year follow-up. In contrast, positive correlations were found in both directions between SOC and SWL in the 10- and 20-year comparison. Thus, one may conclude that SOC appears to be a predictor for SHS and SWL but not for PHS. However, in the 10-year comparison, the predictive effect of health variables (i.e., SHS, PHS, and SWL) on SOC was even stronger than vice versa. A potential explanation for these findings might be the reciprocal relationship between SOC and health outcomes, especially in the medium-term relation. For example, it was reported that the stronger the SOC, the better the perceived quality of life in general (8). Furthermore, SOC seems to be associated with health-enhancing or preserving behavior, e.g., less use of alcohol, being a non-smoker, better oral health care, and better social competence (4, 38).

Overall with regard to the second research question, our data of the 10-year analyses showed that SHS, PHS, and SWL at baseline predicted SOC at follow-up better than vice versa. This may indicate that the previously reported relationship between SOC and health is reciprocal. Within a group of chiropractors, Alcantara et al. (39) were able to confirm the predictive power of mental and physical health for SOC through a random forest analysis. They even found evidence that despite low physical health scores, a high mental health score predicted a high SOC. The results of the 20-year analyses revealed inconsistent associations between SOC and health outcomes, speaking for the declining importance of previous health issues on the SOC in the long-term relationship. As of today, to the best of our knowledge, only the predictive effect of SOC on health and not vice versa has been examined, with several studies reporting that SOC seems to be a health resource promoting resilience and the development of a positive subjective state of health. Data from Suominen et al. (5) confirmed that SOC predicts a positive subjective state of health in a Finnish population over a period of 4 years. A 19-year register-based prospective study from Finland showed that a strong SOC at baseline was associated with a reduced risk of psychiatric disorders 19 years later (17). However, conflicting results have also been reported. For instance, Kivimäki et al. (16) could not find a predictive longitudinal relationship between SOC and health, and there was no difference in the development of health between individuals with a high SOC vs. those with a moderate or low SOC.

Despite empirical findings supporting the importance and validity of the salutogenic theory, there is a lack of knowledge about how SOC develops over time, and how it could be strengthened by interventions or even lowered by crucial life events (40). Some researchers assumed that there is a complex empowerment process characterized by both challenges and a certain willingness of an individual to solve problems (30, 36). It is conceivable that this empowerment process can be supported by good health status. In today's Western societies, the economic status of the general population is at a relatively higher level compared to previous times and other populations (i.e., developing countries). Therefore, individuals may have good internal healing resources, i.e., the potential for active adaptation to new circumstances. This makes it possible for persons to maintain better health for longer periods, which in turn may influence the SOC itself over time. Kase et al. (41) suggested that improving life skills is effective in strengthening SOC. Antonovsky was aware of the impact of social conditions on peoples' health in a society, and he explicitly pointed out the responsibility of the society to create conditions that induce the strengths of coping. Furthermore, he emphasized focusing on structures supporting health rather than on specific risk factors. In addition to programs reducing such risk factors (e.g., obesity, high blood pressure, or smoking), the attention should therefore also be on salutogenic structures of the society. By improving social support or reducing poverty, it is possible to reduce the risk of disease in the long term (42). Therefore, the original definition of SOC as an individual global orientation of the world should be broadened to wider levels like family and community coherence (43).

Our study should be interpreted in light of its strengths and limitations. The main strength of this study is the longitudinal, population-based design with a long follow-up of 20 years. As previous research has mainly utilized cross-sectional study designs, our study may be among the first to report the longitudinal associations between SOC and both self-reported (i.e., SHS, SWL) and objective health outcomes assessed by a physician based on a medical examination (i.e., PHS). The main limitations of the study pertain to the use of questionnaires which may be prone to recall or social desirability bias, albeit all questionnaires used in this research are validated and have been used in large-scale research before. Further studies should therefore also consider hard outcomes (e.g., mortality). Furthermore, in addition to the orthopedic, neurological, and cardiovascular criteria mentioned in this study, it would be recommended to expand the medical examination to include additional clinical parameters such as baseline biomarkers or information about chronic diseases.

In addition, as in any observational study, we cannot draw any conclusion about cause and effect. Nevertheless, the cross-lagged model is a simple and powerful method to test reciprocal effects and has therefore been widely used. However, the application of the cross-lagged model has also been criticized as the cross-lagged estimates conflate between-person and within-person processes, and thus the results may not represent the actual within-person relationship over time [e.g., (44)]. To address this limitation, Hamaker et al. (45) proposed random intercepts cross-lagged model (RI-CLPM) as an alternative. This model allows testing the reciprocal effects within individuals by the inclusion of a latent variable that represents a time-invariant trait-like factor. Since we used ANOVAs to examine between-person and within-person processes in our study, the cross-lagged model was used as an additional analysis where predicting the outcome was the main analytic purpose. However, future studies should include this aspect.

Furthermore, there are other concepts contributing to positive health development, e.g., resilience and empowerment which we did not consider in our analyses. It must be noted that these concepts may be related to SOC and our reported findings. Because of participant attrition, we compared baseline parameters between participants with available data at 20-year follow-up (*n* = 240) and participants who were missing at follow-up (*n* = 152). We found that participants missing at follow-up had a slightly higher (*t* = 1.98, df = 257.55; *p* = 0.049) baseline SOC score (*M* = 60.68, *SD* = 7.86) compared to participants with available data at 20-year follow-up (*M* = 59.19, *SD* = 6.03). Thus, a possible impact of survival bias on the increase in SOC over time in our sample is unlikely. Likewise small but statistically significant differences between respondents and non-respondents were found for SES (χ^2^ = 8.26; df = 3; *p* = 0.041) and physical activity level (χ^2^ = 6.59; df = 1; *p* = 0.010), but not for sex (χ^2^ = 0.190; df = 1; *p* = 0.663), age (χ^2^ = 36.45; df = 29; *p* = 0.161), SHS (*t* = −0.38; df = 377; *p* = 0.707), PHS (*t* = 0.27; df = 268.79; *p* = 0.787) and SWL (*t* = −0.86; df = 382; *p* = 0.389). At baseline, 9.4% of non-responder belonged to the low SES group, 23.5% to the high SES group and 56.7% engaged in sports whereas only 4.7% of responder belonged to a low SES, but 34.3% to a high SES and 69.5% engage in sports at baseline. A potential influence of SES needs to be considered, as our study was conducted in a predominantly Caucasian community in a rather wealthy town in South-Western Germany, as reflected in the comparatively high SES in the demographic data of the sample. Especially since there is evidence that SES is positively related to SOC (46) and seems to have an impact on health and well-being (47). Gomes et al. (48) postulated that SES is an important predictor of health behavior and health-related quality of life. Other results indicated that deficits in health and social resources were largely responsible for the precipitous decline in SOC after age 70. When controlling for these deficits, SOC increased continuously into advanced old age (49). An element of selection bias may therefore be present and should be considered when interpreting results. Thus, findings may not be generalizable to predominantly urban areas or larger cities, as well as to communities with a higher frequency of persons with migration backgrounds and/or less wealthy communities.

We provided evidence of reciprocal longitudinal associations between SOC and both self-reported and physician-assessed health in community-dwelling adults aged ≥33 years. The fact that our study could show that the SOC is not only a protective factor for health in later life, but also significantly decreases with previous health issues (in the medium-term relation of 10 years), can be interpreted as a need to strengthen individuals concepts of life after crucial health issues and to emphasize in general the importance of mental health in current Western societies. More research is needed to confirm our findings and to further untangle the direction of associations between SOC and health in adults.

## Data Availability Statement

The raw data supporting the conclusions of this article will be made available by the authors, without undue reservation.

## Ethics Statement

The studies involving human participants were reviewed and approved by Ethics Committee of the Karlsruhe Institute of Technology (KIT). The patients/participants provided their written informed consent to participate in this study.

## Author Contributions

AD, JK-R, and SS analyzed and interpreted the data. AD prepared the draft manuscript, while JK-R, SS, KB, and AW provided the critical revisions. All authors contributed to the article and approved the submitted version.

## Funding

The authors did receive specific funding from AOK Mittlerer Oberrhein and the community of Bad Schönborn for this research.

## Conflict of Interest

The authors declare that the research was conducted in the absence of any commercial or financial relationships that could be construed as a potential conflict of interest.

## Publisher's Note

All claims expressed in this article are solely those of the authors and do not necessarily represent those of their affiliated organizations, or those of the publisher, the editors and the reviewers. Any product that may be evaluated in this article, or claim that may be made by its manufacturer, is not guaranteed or endorsed by the publisher.

## Abbreviations

GRR: generalized resistance resources
PHS: physical health status
SHS: self-rated health status
SOC: sense of coherence
SWL: satisfaction with life.
